# Overexpression of P16 reversed the MDR1-mediated DDP resistance in the cervical adenocarcinoma by activating the ERK1/2 signaling pathway

**DOI:** 10.1186/s13008-019-0048-6

**Published:** 2019-07-06

**Authors:** Yun Xiao, Mei-rong Liang, Cheng-cheng Liu, Ya-nan Wang, Yang Zeng, Jun Zhou, Hui-ting Zhu, Qin Wang, Yang Zou, Si-yuan Zeng

**Affiliations:** 1 Medical College of Nanchang University, No.461, Bayi Street, Nanchang, 330006 Jiangxi China; 2 Department of Radiotherapy, Jiangxi Tumor Hospital, No. 519, Beijingdong Street, Nanchang, 330029 Jiangxi China; 3 Department of Oncology, Jiangxi Maternal and Child Health Hospital, No. 318, Bayi Street, Nanchang, 330006 Jiangxi China; 4 Center Laboratory, Jiangxi Maternal and Child Health Hospital, No. 318, Bayi Street, Nanchang, 330006 Jiangxi China; 5 Department of Obstetrics and Gynecology, Duchang Maternal and Child Health Hospital, No. 79, Dongfeng Street, Duchang, 332600 Jiangxi China

**Keywords:** Cervical adenocarcinoma, Cisplatin (DDP), P-glycoprotein, ERK1/2, pERK1/2, Multidrug resistance protein 1 (MDR1)

## Abstract

**Background:**

To investigate the role of P16 (INK4a)-extracellular signal related kinase 1/2 (ERK1/2) signaling pathway in cisplatin (DDP) resistance induced by multidrug resistance protein 1 (MDR1), also known as P-glycoprotein (P-gp), in cervical adenocarcinoma.

**Methods:**

A human DDP-resistant HeLa cell line (HeLa/DDP) was constructed using the combination of incremental and intermittent administration of DDP. Cell Counting Kit-8 (CCK-8) assay was used to measure the IC50 and resistance index (RI) of cells. The morphological changes and population doubling time were observed under an inverted microscope. Plate cloning formation assay was performed to evaluate the cell proliferation and tumorigenic ability. Cell invasion and migration were determined by transwell assays. Besides, the expression of P16, phosphorylated extracellular signal related kinase 1 and 2 (pERK1/2), total ERK1/2 and MDR1 were measured using western blot analysis. The ERK-specific inhibitor U0126 and agonist TPA was used to explore the role of ERK.

**Results:**

The DDP-resistant cervical adenocarcinoma HeLa/DDP cell line was successfully established, which showed stronger cell growth, invasion, and migration. In the HeLa/DDP cells, pERK1/2 was downregulated, P-gp was upregulated and P16 was downregulated. Overexpression of P16 led to a significant decrease in the proliferation rate, migration ability, and invasion ability of the HeLa/DDP cells. Furthermore, overexpression of P16 increased and the decreased expression of pERK1/2 and P-gp in the HeLa/DDP cells, respectively. Treatment of HeLa/DDP cells transfected with P16 plasmid with ERK-specific inhibitor U0126 significantly decreased the expression of pERK1/2 and increased the expression of P-gp from 6 h to 48 h. Moreover, after 72 h, the expression of pERK1/2 was up-regulated and the expression of P-gp was inhibited.

**Conclusion:**

Overexpression of P16 could partially reverse the MDR1-mediated DDP resistance in the cervical adenocarcinoma by the enhancement of phosphorylation of ERK signaling pathway, which provided a theoretical basis for the treatment of DDP resistance in cervical adenocarcinoma.

**Electronic supplementary material:**

The online version of this article (10.1186/s13008-019-0048-6) contains supplementary material, which is available to authorized users.

## Background

Chemotherapy is the most common treatment for cancer. Cervical adenocarcinoma is not sensitive to radiotherapy, mainly due to the multidrug resistance (MDR) of tumor cells. Therefore, a new treatment strategy for cervical adenocarcinoma is in urgent need.

Many studies showed that MDR was associated with the MDR1/P-glycoprotein (P-gp) and that MDR1 was overexpressed in several drug-resistant cell lines and cervical cancer cells [1, 2]. They also demonstrated that several structural and functional lipophilic antineoplastic agents, including cisplatin (DDP), induced MDR1 expression [1, 3]. DDP is a nonspecific anticancer drug, which is widely used in the treatment of human solid tumors, such as cervical adenocarcinoma [1, 2]. DDP induces apoptosis by activating various signaling pathways, including ATR (ATM and RAD3-related), P53, P73 and mitogen-activated protein kinase (MAPK) [1]. However, DDP-induced apoptosis weakens over time, resulting in drug resistance, which is a major problem in chemotherapy [3]. In addition to the reduced apoptosis, studies showed that DDP resistance was associated with the repair of DNA damage. DDP resistance led to the overexpression of human epidermal receptor 2 (HER-2/NEU) and B-cell lymphoma-2 (Bcl-2), loss of P53 function, caspase inactivation, increased EGFR activity, and activation of the P13K/AKT/extracellular signal related kinase (ERK) pathway [3, 4].

A number of studies have suggested the important role of ERK1/2 in resistance to chemotherapy drugs, and that activation of the ERK protein was positively correlated with tumor cell resistance [5, 6]. Substrates of ERK1/2 include the transcription factors Ets-like protein 1, artificial transcription factor, nuclear factor-κB (NF-κB), activator protein 1, c-fos, and c-Jun [7]. NF-kB, c-fos, and c-jun have been shown to be associated with transcriptional activation of the MDR1 gene [6, 7]. A previous study also demonstrated the high expression of P16 (INK4a) in cervical squamous cell carcinoma and cervical adenocarcinoma [7]. In addition, it has been reported that P16 was involved in tumor cell resistance to artesunate [8]. Furthermore, the expression of P16 in ovarian epithelial carcinoma was positively correlated with the expression of MDR1, suggesting that P16 was associated with tumor cell resistance [9]. P16 may regulate the expression of the MDR1 gene and thus mediate tumor cell resistance via the ERK1/2 signaling pathway [6–9].

The relationship between P16 and DDP resistance in cervical adenocarcinoma is unclear, and there have been no studies about the role of ERK in the MDR1-mediated DDP resistance in DDP-resistant cervical adenocarcinoma cells. In the present study, we established a DDP-resistant cervical adenocarcinoma cell line, HeLa/DDP, and then explored the expression of p16, phosphorylated ERK1/2 (pERK1/2), ERK1/2 and MDR1 in HeLa and HeLa/DDP cells. In addition, we clarified the molecular mechanism underlying the role of the P16-ERK1/2 signaling pathway in DDP resistance in cervical adenocarcinoma.

## Materials and methods

### Establishment of a DDP-resistant cervical adenocarcinoma cell line

The cervical adenocarcinoma HeLa cell line was provided by the Central Laboratory of Jiangxi Maternal and Child Health Hospital. HeLa cells were incubated in RPMI 1640 medium containing 10% fetal bovine serum (FBS) and 100 U/l of penicillin and streptomycin and then placed in an incubator at 37 °C, 5% CO_2_ and saturated humidity. And the cells were passaged by using trypsin digestion containing 0.02% EDTA. Inoculation was induced in vitro using a combination of incremental and intermittent administration of cisplatin (DDP). DDP was dissolved in phosphate buffered saline (PBS) buffer. The initial concentration of DDP in the culture was 0.01 μM and the concentration was increased to 8 μM. The treatment was repeated four times and 3 days each time within 4–7 weeks. Taking the cell repair between cell cycles into account, the drug dose was doubled after four cycles and repeated until the highest concentration. After about 10 months, a DDP-resistant cervical adenocarcinoma cell line (HeLa/DDP) was established. The cell line was authenticated by STR DNA fingerprinting in Sangon Biotech (Shanghai) Co., Ltd (Shanghai, China).

### Cell Counting Kit-8 (CCK-8) assay

Hela and HeLa/DDP cells in logarithmic growth phase were inoculated into 96-well plates (2 × 10^3^ per well). After 24 h of culture, the cells were treated with DDP by doubling dilution. The blank group (without cells and DDP) and control group (without DDP) were included. All the experiments were performed in triplicate. After 72 h of incubation, 100 μl of fresh medium was added. After another 2 h, the absorbance was then measured at 490 nm.

### Observation of cell morphology and detection of population doubling times

Morphological changes in Hela and Hela/DDP cells were observed under an inverted microscope. Single cell suspensions of HeLa and Hela/DDP cells from the logarithmic growth phase were seeded into 24-well plates with 1 × 10^4^ cells per well (in triplicate). The cells were counted every day for 7 days after culture, and the average was taken. After 7 days, curve fitting was performed with the function-fitting model of SPSS software.

### Plate cloning formation assay

HeLa and Hela/DDP cells were seeded into six-well plates (10^3^ cells per well). HeLa, HeLa/DDP, HeLa + DDP and HeLa/DDP + DDP groups were established. The cells were treated with 8 μM DDP and then incubated for 2 weeks at 37 °C in a 5% CO_2_ incubator. The cloning formation rate (%) was expressed as (number of clones/number of inoculated cells) × 100%.

### Transwell assays

#### Invasion assay

Matrigel gel, which had been melt into a liquid, was mixed with the serum-free medium in a 1:8 ratio and 50 μl of which were taken for per well. The wells were placed in a 5% CO_2_ incubator overnight at 37 °C. Then, 200 μl of cell suspension (1 × 10^5^/ml) were added to the upper chamber, and 500 μl medium containing 10% FBS was added to the lower chamber. After incubated in a 5% CO_2_ incubator for 48 h, the cells in the lower layer of the microporous membrane were counted with an optical microscope (100×), and the mean values were determined by counting 10 randomly selected fields. All the experiments were performed in triplicate.

#### Migration assay

The cells were incubated for 24 h in a 5% CO_2_ incubator. They were then stained with crystal violet and photographed under an optical microscope (100×). The numbers of cells in the lower layer of the microporous membrane were counted in 10 randomly selected fields, and the mean values were calculated. The experiments were performed in triplicate.

### Reverse transcription and quantitative real-time PCR

Total RNA extracted from HeLa and Hela/DDP cells using TRIzol (Invitrogen) was reverse-transcribed to the first-strand cDNA. Quantitative real-time PCR (qPCR) was performed on StepOnePlus™ real-time PCR system (Applied Biosystems™). The qPCR conditions were as follows: 95 °C, 30 s and 40 cycles of 95 °C, 5 s, 60 °C, 30 s. Quantification was performed by using the comparative Ct (ΔΔCt) method according to manufacturer’s instructions. The primers used for qPCR are listed below:

**Table:** 

P16-F	CTTCCTGGACACGCTGGT
P16-R	GCATGGTTACTGCCTCTGGT
GAPDH-F	ACCCCTTCATTGACCTCAAC
GAPDH-R	CATCGCCCCACTTGATTTTG

### Western blot analysis

Total protein was extracted from the HeLa and HeLa/DDP cells. The protein concentration was measured by bicinchoninic acid (BCA) and 30 μg protein Page: 8 was loaded into a polyacrylamide gel for sodium dodecyl sulfate-polyacrylamide gel electrophoresis. After wet transfer, the nitrocellulose membrane was blocked with 5% skim milk for 40 min, followed by being incubated overnight at 4 °C with the primary antibody specific for P16 (Proteintech, US), P-gp (Abcam, US), pERK1/2 (Cell Signaling Technology, US), total ERK1/2 (Cell Signaling Technology, US) and β-actin (Chinese fir golden bridge company, China). The next day, the film was washed with Tris Buffered Saline Tween (TBST) buffer 15 min for three times and then incubated with horseradish peroxidase-labeled goat anti-rabbit IgG (CWBIO, China) or goat anti-mouse IgG (TransGen, Beijing, China) secondary antibody at room temperature for 1 h. The film was washed again with TBST buffer and finally stained with an enhanced chemiluminescent agent and exposed to a gel imaging system (Chemi DOCtm XRS, Bio-Rad, Beijing, China).

### Plasmid construction and cell transfection

The construction of pEX-2 P16 plasmid was performed by a manufacturer (Shanghai GenePharma Co., Ltd, China). Transfection of HeLa/DDP cells with the pEX-2 empty vector or P16 plasmid was performed using the lipofectamine™ 2000 transfection reagent according to the manufacturer’s instructions. The pEX-2 vector carries a reporter gene, green fluorescent protein (GFP), whose expression could be used to determine the transfection efficiency.

### Statistical analysis

Quantitative data are shown as mean ± s.e.m. Differences between two groups and more than two groups were analyzed by student *t*-test and two-way analysis of variance, respectively. *P* values less than 0.05 were considered as statistically significant.

## Results

### Comparison of the proliferation and tumorigenic ability of HeLa and HeLa/DDP cells

**Fig. 1:**
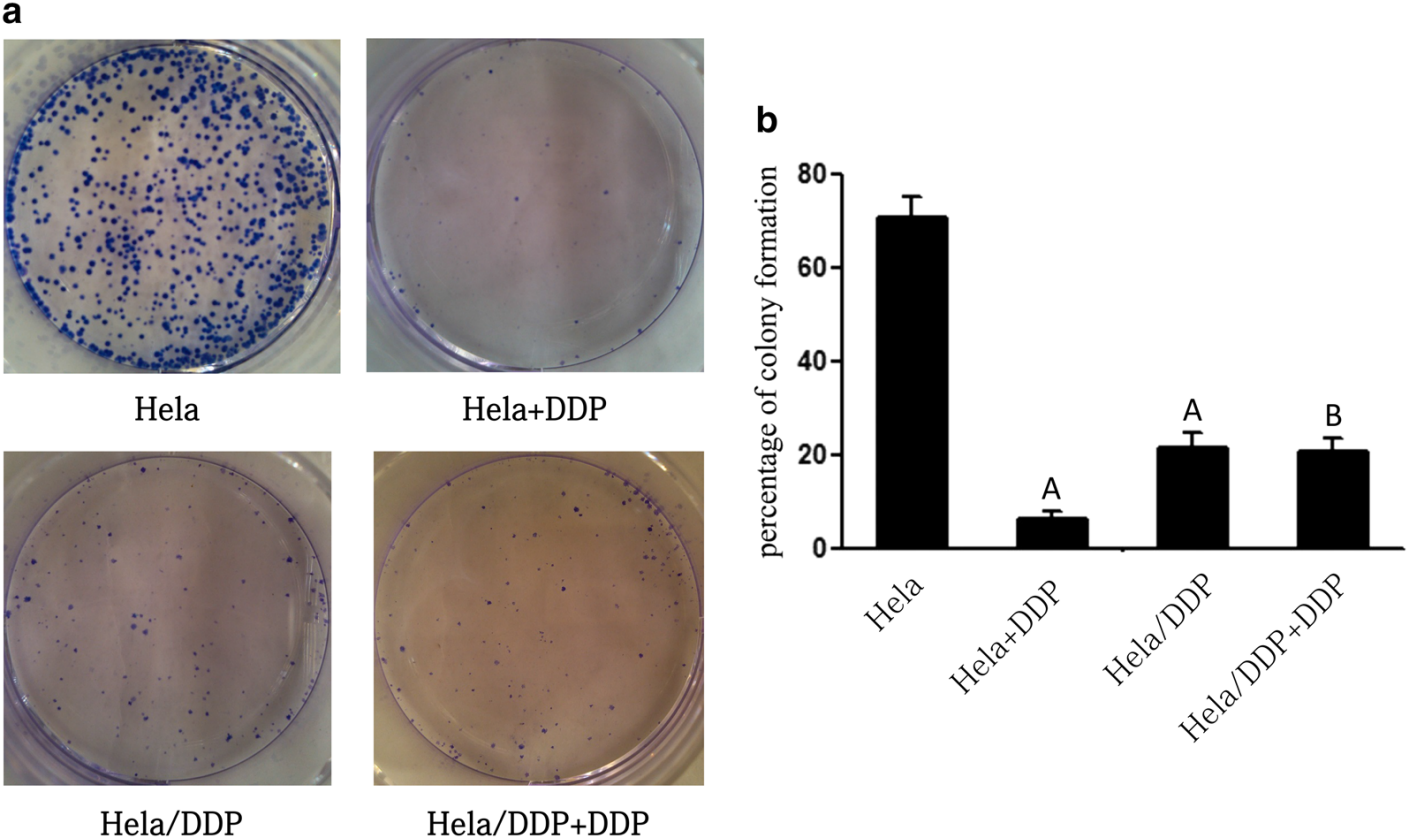
Cell proliferation and tumorigenic ability of HeLa and HeLa/DDP. **a** Representative images of flat colonies. **b** The cloning formation rates of different groups. Values represent the mean ± S.E.M. A, *P* < 0.05 versus the HeLa group (Student’s *t*-test); B, *P* < 0.05 versus the HeLa+DDP group (Student’s *t*-test)

A DDP-resistant cervical adenocarcinoma cell line (HeLa/DDP) was successfully established by exposing the cells to increased concentrations of DDP at intermittent intervals (Additional file 1: Figure S1). According to the CCK-8 assay, the IC50 of DDP in the HeLa cells and HeLa/DDP cells was 1.72 and 6.37 μg/ml, respectively, which was statistically significant (*P *= 0.001) (Additional file 2: Figure S2). And the drug resistance index (RI) of HeLa/DDP cells to DDP was 3.7. The population doubling times of the HeLa cells and HeLa/DDP cells were 38 h and 63 h, respectively (Additional file 3: Figure S3). The cloning formation rate in the HeLa group was significantly higher than that in HeLa/DDP group (70.9 ± 4.21% vs 21.5 ± 3.59%; *P *< 0.01), indicating that the proliferation of HeLa cells was higher than that of HeLa/DDP cells in the absence of DDP. Further, the colony formation rate in the HeLa + DDP group and HeLa/DDP + DDP group were 6.5 ± 1.38% and 20.8 ± 2.94%, respectively, suggesting that the proliferation of HeLa cells was lower than that of the HeLa/DDP cells in the presence of DDP. Regardless of the DDP treatment, there was no significant difference in the colony formation rate of HeLa/DDP cells, indicating that the HeLa/DDP cells showed resistance to DDP (Fig. 1).

### Comparison of the invasion and migration of HeLa and HeLa/DDP cells

**Fig. 2:**
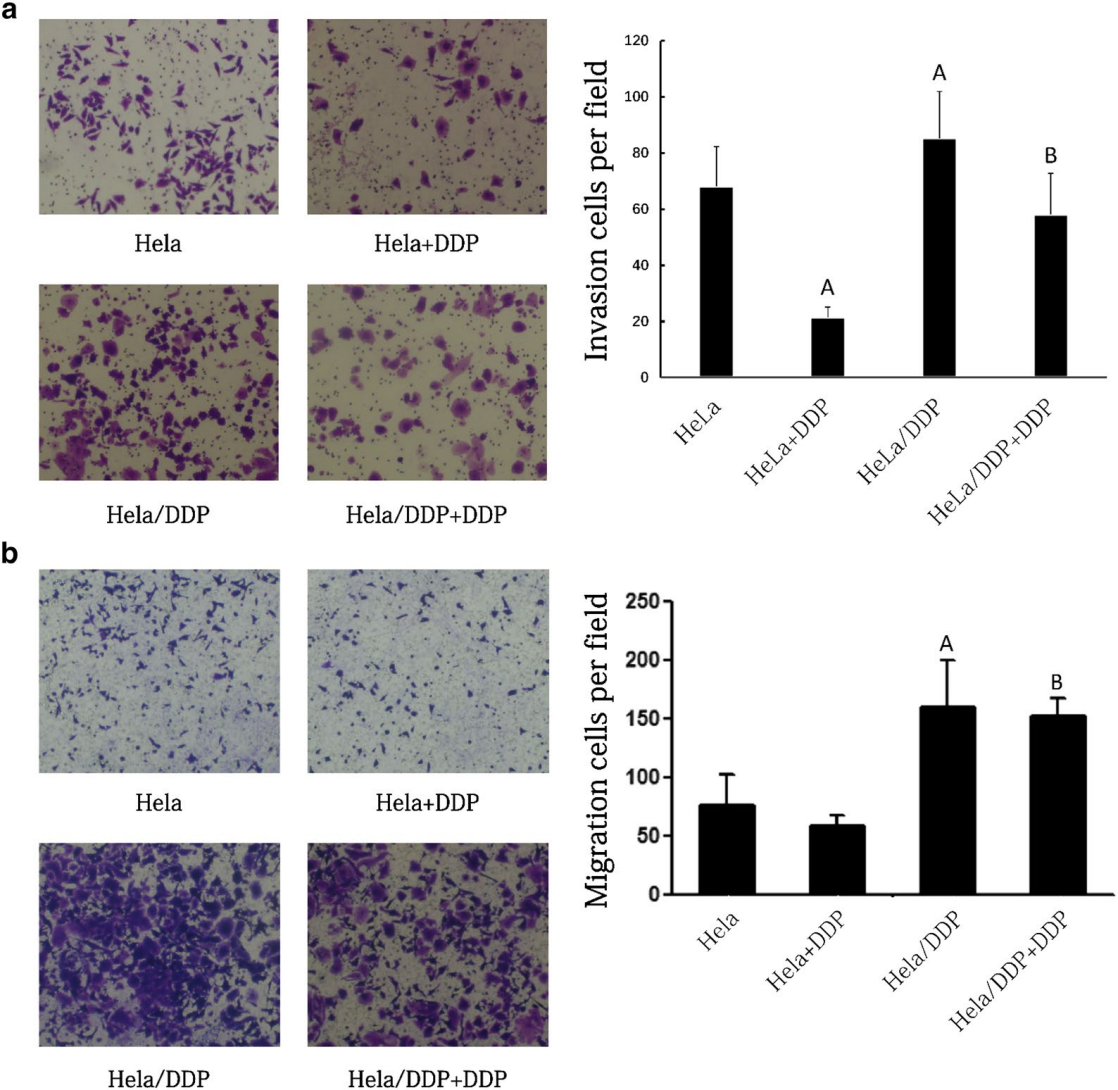
Comparison of the invasion (**a**) and migration (**b**) of HeLa and HeLa/DDP cells. A, *P* < 0.05 versus the HeLa group (Student’s *t*-test); B, *P* < 0.05 versus the HeLa+DDP group (Student’s *t*-test)

The invasion ability of the HeLa/DDP group was significantly higher as compared to that of the HeLa group (85.3 ± 16 vs 68.2 ± 14; *P* < 0.05). After DDP treatment, the invasion ability was significantly decreased in HeLa cells (21.5 ± 3.6) compared with HeLa/DDP cells (58.1 ± 14.6) (*P* < 0.0001). Importantly, HeLa cells showed a stronger invasion ability relative to the HeLa + DDP group (*P* = 0.005). However, there was no significant difference in the invasion ability between the HeLa/DDP group and HeLa/DDP + DDP group (*P* > 0.05). Taken together, DDP significantly weakened the invasion ability of HeLa cells without significantly changing the invasive ability of HeLa/DDP cells (Fig. 2a).

The migration ability of HeLa/DDP cells was significantly stronger than that of HeLa cells (160.1 ± 37 vs 76.1 ± 26; *P* < 0.0001). In the presence of DDP, the migration ability of HeLa/DDP group was also significantly stronger than that of HeLa/DDP group (59.4 ± 8.6 vs 153 ± 15; *P* < 0.0001). However, there was no statistically significant change in the migration ability of HeLa cells and HeLa/DDP cells when exposed to DDP (*P* > 0.05) (Fig. 2b).

### Expression of p16, P-gp, and ERK1/2 in HeLa and HeLa/DDP cells

**Fig. 3:**
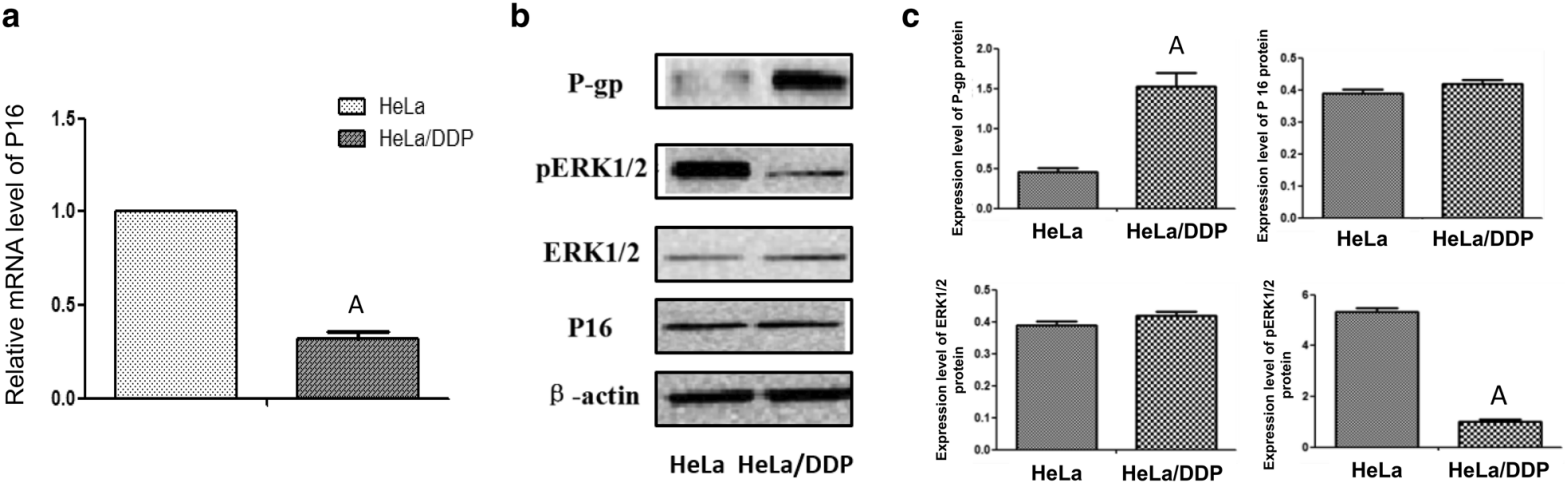
P-gp, pERK1/2, and P16 expressions in the HeLa group and HeLa/DDP group. **a** The P16 mRNA expression in the HeLa group and HeLa/DDP group. **b** Representative images of the protein expressions of P-gp, ERK1/2, pERK1/2, and P16 in the HeLa group and HeLa/DDP group by Western blot. **c** Quantitative analysis of the protein expression of P-gp, ERK1/2, pERK1/2, and P16 relative to that of β-actin. Values represent the mean ± S.E.M. A, *P* < 0.05 versus the HeLa group (Student’s *t*-test)

The mRNA expression of P16 in HeLa/DDP cells was significantly lower as compared to that of the HeLa cells, however, its protein levels were relatively the same in both HeLa and HeLa/DDP cells (Fig. 3). The expression of P-gp protein was significantly higher in HeLa/DDP cells compared with HeLa cells. In contrast, the expression of pERK1/2 protein was significantly lower in HeLa/DDP cells than that in HeLa cells. There was no significant difference in total ERK1/2 protein levels between HeLa and HeLa/DDP cells (Fig. 3).

### Effects of overexpression of P16 on the cell proliferation, invasion and migration of HeLa/DDP cells

**Fig. 4:**
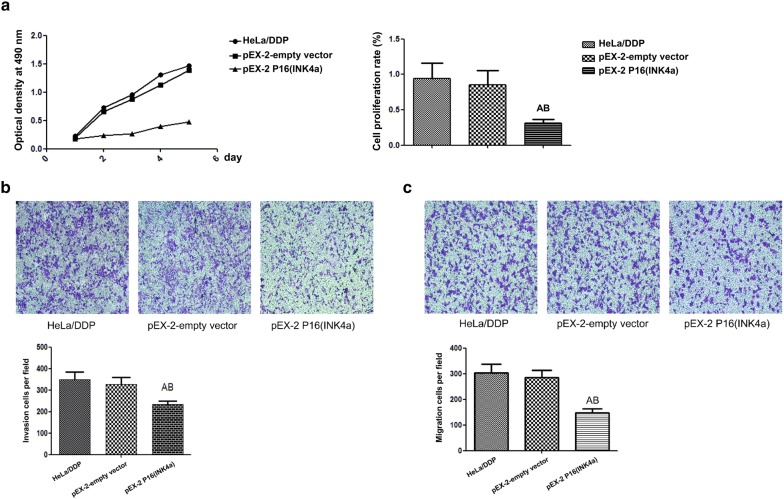
Effects of P16 overexpression on the cell proliferation (**a**), invasion (**b**), and migration (**c**) of HeLa/DDP cells. Values represent the mean ± S.E.M. Magnification, ×100. A, *P* < 0.05 versus HeLa/DDP (Student’s *t*-test); B, *P* < 0.05 versus pEX-2-empty vector (Student’s *t*-test)

The pEX-2 empty vector or P16 plasmid was transfected into HeLa/DDP cells, and the expression of GFP was observed 24 h after transfection (Additional file 4: Figure S4). Cell growth analysis showed that there was no significant difference in the cell proliferation rate between HeLa/DDP cells and HeLa/DDP cells transfected with empty vector. However, overexpressing P16 led to a significant decrease in the proliferation rate of HeLa/DDP cells, indicating that the overexpression of P16 inhibited the growth of HeLa/DDP cells (Fig. 4a).

Moreover, as shown in Fig. 4b, the invasion ability was significantly lower in HeLa/DDP cells transfected with P16 than that in empty vector and HeLa/DDP groups (*P *< 0.05). Consistent with this, the cell migration ability was significantly decreased in HeLa/DDP cells transfected with P16 compared with the normal control group and negative control group (*P* < 0.05), as depicted in Fig. 4c. Therefore, overexpression of P16 significantly decreased the invasion and migration of HeLa/DDP cells.

### Effects of P16 overexpression on the expression of pERK1/2 and P-gp

**Fig. 5:**
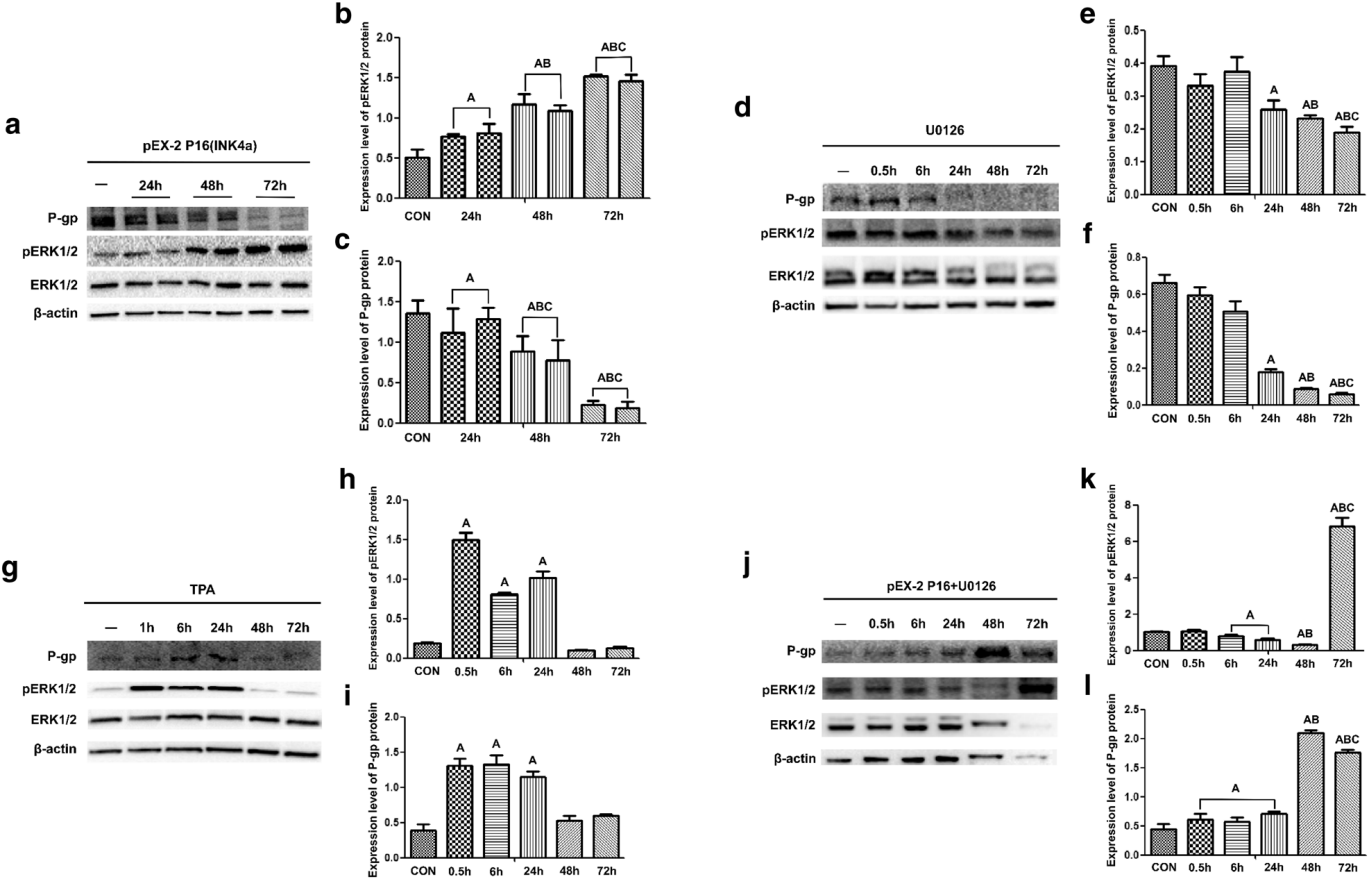
Analysis of the expression of ERK1/2, pERK1/2, and P-gp by Western blot. **a**–**c** The expression of pERK1/2 and P-gp in HeLa/DDP cells after transfection with pEX-2 empty vector or P16 plasmid. **a** Representative images of ERK1/2, pERK1/2, and P-gp. **b** Quantitative analysis of pERK1/2 protein expression relative to that of total ERK1/2. **c** Quantitative analysis of P-gp expression relative to that of β-actin. **d**–**f** Effects of U0126 on the expression of pERK1/2 and P-gp in HeLa/DDP cells at different time points. **d** Representative images of ERK1/2, pERK1/2, and P-gp. **e** Quantitative analysis of the pERK1/2 protein expression relative to total ERK1/2 expression. **f** Quantitative analysis of the P-gp expression relative to β-actin expression. **g**–**i** Effects of TPA on the expression of pERK1/2 and P-gp in HeLa/DDP cells at different time points. **g** Representative images of ERK1/2, pERK1/2, and P-gp. **h** Quantitative analysis of the pERK1/2 protein expression relative to total ERK1/2 expression. **i** Quantitative analysis of the P-gp expression relative to β-actin expression. **j**–**l** Effects of U0126 on the expression of pERK1/2 and P-gp in HeLa/DDP cells after P16 transfection at different time points. **j** Representative images of ERK1/2, pERK1/2, and P-gp. **k** Quantitative analysis of the pERK1/2 protein expression relative to total ERK1/2 expression. **l** Quantitative analysis of the P-gp expression relative to β-actin expression. A, *P* < 0.05 versus CON (control) group; B, *P* < 0.05 versus 24 h group; C, *P* < 0.05 versus 48 h group

The protein expression was detected 24 h, 48 h, and 72 h after HeLa/DDP cells transfected with the pEX-2 P16 plasmid. The results showed that the expression of pERK1/2 protein in the transfected group was significantly higher than that in the blank control group, whereas the expression of P-gp was significantly lower (*P* < 0.05) (Fig. 5a–c).

### Effects of the ERK-specific inhibitor U0126 and agonist TPA on the expression of P-gp in HeLa/DDP cells

The protein expression was detected at different time points after HeLa/DDP cells treated with U0126 (50 µM) or TPA (150 nM). Twenty-four hours after treatment, U0126 led to a time-dependent decrease in the protein levels of pERK1/2 and P-gp in the HeLa/DDP cells (Fig. 5d–f). Treatment of cells with TPA resulted in a significant rise in the expression of pERK1/2 and P-gp from 1 h to 24 h, however, their expression was decreased after 48 h (Fig. 5g–i).

The HeLa/DDP cells transfected with the pEX-2 P16 plasmid for 72 h were treated with 50 µM U0126 for the different time period. The expression of pERK1/2 was significantly decreased by U0126 from 6 h to 48 h. Moreover, the expression of P-gp was gradually increased after U0126 treatment from 0.5 h to 48 h. However, with the increase of time, the expression of pERK1/2 was up-regulated and the expression of P-gp was inhibited after 72 h of U0126 treatment (Fig. 5j–l).

## Discussion

The generation of DDP-resistant cervical adenocarcinoma cell line is evidence-based [10, 11]. Two previous methods were used to establish drug-resistant tumor cell lines: intermittent induction using high-dose drugs and incremental induction using gradient concentrations of drugs [12, 13]. In the present study, we successfully established a DDP-resistant adenocarcinoma cell line (HeLa/DDP) via the combination of incremental and intermittent administration of DDP. This model is ideal for further studies at the cellular level to elucidate the molecular mechanism of DDP resistance in cervical adenocarcinoma.

It has been reported that sensitivity to chemotherapy was negatively correlated with doubling time, which means a shorter or longer doubling time was associated with enhanced or decreased chemosensitivity, respectively [14]. In the present study, the population doubling time of HeLa/DDP cells was longer than that of HeLa cells, suggesting that HeLa/DDP cells were less sensitive to chemotherapy. The IC50 of DDP in HeLa/DDP cells and HeLa cells was 6.37 and 1.72 μg/ml, respectively, with the RI of HeLa/DDP cells, was as high as 3.7, and drug resistance was stable.

According to the National Cancer Institute, invasion and migration are the main causes of mortality in malignancies, and drug-resistant cell lines show stronger invasion and migration abilities than parental cells [15]. In the present study, the migration and invasion abilities of HeLa/DDP cells were enhanced compared to those of HeLa cells, which may be related to the expression of a number of factors. Zhang et al. found that P-gp induced tyrosine phosphorylation of annexin A2, which promoted the invasiveness of resistant breast cancer cells [15]. Lu et al. reported that overexpression of miR-29a significantly inhibited the invasive ability of oral squamous cell carcinoma. In contrast, down-regulation of miR-29a promoted the invasion of oral squamous cell carcinoma cells and tumor cell resistance [16]. Zhao et al. found that inhibition of Fanconi anemia complementation group F protein increased the sensitivity of breast cancer cells to doxorubicin and resulted in decreased migration and invasion of tumor cells [17]. The epithelial mesenchymal transition (EMT) was also reported to be associated with drug resistance [17]. Both the invasion and migration abilities of cells with an EMT phenotype were enhanced in gemcitabine-resistant pancreatic cancer cell lines. And when the EMT was partially reversed, the expression of platelet-derived growth factor-D and hypoxia-inducible factor-1a was downregulated [13, 17, 18]. It has also been reported that high mobility group box-1 protein (HMGB1) increased the expression of P-gp, thereby promoting drug resistance and the proliferation and invasion of tumor cells [19]. Further studies are needed to explore the potential role of the above factors in the increased invasion and migration of the HeLa/DDP cells found in the present study.

A previous study showed that MDR1 was involved in the regulation of cell invasion and migration via its interaction with other proteins, which resulted in the activation of ERK1/2 and P38 MAPK signaling pathways and the induction of tumor cell invasion-related proteins, such as matrix metalloproteinases [20]. In the present study, the expression of MDR1 was significantly higher in HeLa/DDP-resistant cells than that in HeLa cells, suggesting that long-term stimulation of DDP could lead to elevated MDR1 expression in HeLa cells, resulting in increased intracellular drug excretion and decreased intracellular drug concentrations, and leading to drug resistance of HeLa cells. Notably. it is not clear whether levels of MDR1 in Hela and Hela/DDP cells lines are changing after cisplatin exposure, which would be considered in our future work.

The ERK1/2 pathway was involved in various processes, including cell growth, proliferation, and differentiation. Numerous studies have reported that the overactivation of ERK1/2 appears to be positively correlated with tumor cell resistance to chemotherapy with the potential mechanism involving the regulation of drug-resistant gene expression [5, 21–23]. Xiao et al. reported that increased expression of RAF1/ERK, pERK and MDR protein were associated with drug resistance of pancreatic cancer cells to chemotherapy [24]. Many studies found that exposure to DDP led to activation of ERK1/2 [24]. However, some studies reported that ERK activity was reduced in drug-resistant tumor cells [25]. Alakananda et al. demonstrated that the expression levels of ERK2 were decreased in DDP-resistant HeLa cells and that inhibition of ERK activity by the ERK-specific inhibitor U0126 attenuated the cytotoxicity of DDP to cells, which may result from the degradation of p53 and activation of protein kinase C in HeLa cells by a papillomavirus [22]. In this study, we found that pERK1/2 expression was downregulated and P-gp expression was upregulated in DDP-resistant cervical adenocarcinoma cells, suggesting that inhibition of the ERK1/2 pathway may play regulatory roles in the resistance of cervical adenocarcinoma cells.

P16, an important negative regulator of the G1 phase, is directly involved in the cell cycle regulation. The role of P16 as a tumor suppressor gene in most tumors is well known [26–29]. In the present study, the mRNA expression of P16 was downregulated in HeLa/DDP cells, however, its protein expression was not affected, which is might due to the specificity of the P16 antibody and post-transcriptional regulation. Upregulation of P16 significantly inhibited cell growth, invasion, and migration of HeLa/DDP cells, suggesting that P16 may play an important role in the inhibition of DDP resistance in cervical adenocarcinoma cells.

It has been reported that the regulation of P15 (INK4b) and P16 (INK4a) mRNA and protein levels in HepG2 cells was mediated by the ERK signaling pathway [30]. In contrast, some studies also suggest that P16 affects the expression of MDR1 by regulating the ERK signaling pathway [23, 31]. In the present study, overexpression of P16 decreased and increased the expression of P-gp and pERK1/2, respectively.  Furthermore, the HeLa/DDP cells transfected with P16 were treated with ERK-specific inhibitor U0126, which led to a downward trend in pERK expression and an upward trend in P-gp expression over time. Moreover, with the increase of time, the expression of pERK was up-regulated, while the expression of P-gp was simultaneously down-regulated. Taken together, these results showed that in the cells overexpressing P16, U0126 specifically blocked the phosphorylation of ERK, which increased the expression of P-gp in a time-dependent manner and when this inhibition was removed, the expression of P-gp was decreased at the same time. All these results indicated that overexpression of P16 might inhibit the expression of P-gp through activation of the pERK pathway. Different from this conclusion, it has been shown that the activity of ERK1/2 was enhanced in gemcitabine-resistant intrahepatic cholangiocarcinoma cells, and the use of U0126 may have a synergistic effect on tolerance to gemcitabine [21].

## Conclusion

In this study, we successfully constructed a DDP-resistant human cervical adenocarcinoma cell line. The doubling time, drug resistance, cell growth, invasion and migration abilities were increased in HeLa/DDP cells with the expression of P16, P-gp, and pERK1/2 being downregulated, upregulated, and downregulated, respectively. This study also confirmed that overexpression of P16 partially reversed the MDR1-mediated DDP resistance in the DDP-resistant cervical adenocarcinoma cells by activation of the ERK signaling pathway. All these results provide a theoretical basis for the treatment of DDP resistance in cervical adenocarcinoma.

## Additional files

**Additional file 1: Figure S1.** Morphological observation of HeLa and HeLa/DDP. Magnification, ×100.

**Additional file 2: Figure S2.** IC50 of HeLa and HeLa/DDP in the presence of DDP. Values represent mean ± S.E.M. **P < 0.01 versus HeLa group (Student’s *t*-test).

**Additional file 3: Figure S3.** HeLa and HeLa/DDP population doubling time curves.

**Additional file 4: Figure S4.** The expression of GFP under a fluorescence microscope in HeLa/DDP cells after transfection with pEX-2 for 24 h. A. HeLa/DDP cells transfected with pEX-2 P16 (INK4a) under an inverted microscope. B. HeLa/DDP cells transfected with pEX-2 P16 (INK4a) under a fluorescence microscopy. C. HeLa/DDP cells transfected with pEX-2 empty vector under an inverted microscope. D. HeLa/DDP cells transfected with pEX-2 empty vector under a fluorescence microscopy. Magnification, ×100.

## Data Availability

The data that support the findings of this study are available from the corresponding author upon reasonable request.

## Abbreviations

ERK1/2: extracellular signal related kinase 1/2
MDR1: multidrug resistance protein 1
P-gp: P-glycoprotein
CCK-8: Cell Counting Kit-8
RI: resistance index
pERK1/2: phosphorylated extracellular signal related kinase 1 and 2
HeLa/DDP: DDP-resistant HeLa cell line
DDP: cisplatin
MDR: multidrug resistance
MAPK: mitogen-activated protein kinase
ATR: ATM and RAD3-related
HER-2/NEU: human epidermal receptor 2
Bcl-2: B-cell lymphoma-2
ERK: extracellular signal related kinase
NF-κB: nuclear factor-κB
FBS: fetal bovine serum
PBS: phosphate buffered saline
qPCR: quantitative real-time PCR
ΔΔCt: the comparative Ct
BCA: bicinchoninic acid
TBST: Tris Buffered Saline Tween
GFP: green fluorescent protein
EMT: epithelial mesenchymal transition
HMGB1: high mobility group box-1 protein
