# Supplementary figures and images for: Overexpression of P16 reversed the MDR1-mediated DDP resistance in the cervical adenocarcinoma by activating the ERK1/2 signaling pathway
Source: Cell Div. 2019 Jul 6;14:6. doi: 10.1186/s13008-019-0048-6 (PMC6612198; doi:10.1186/s13008-019-0048-6)

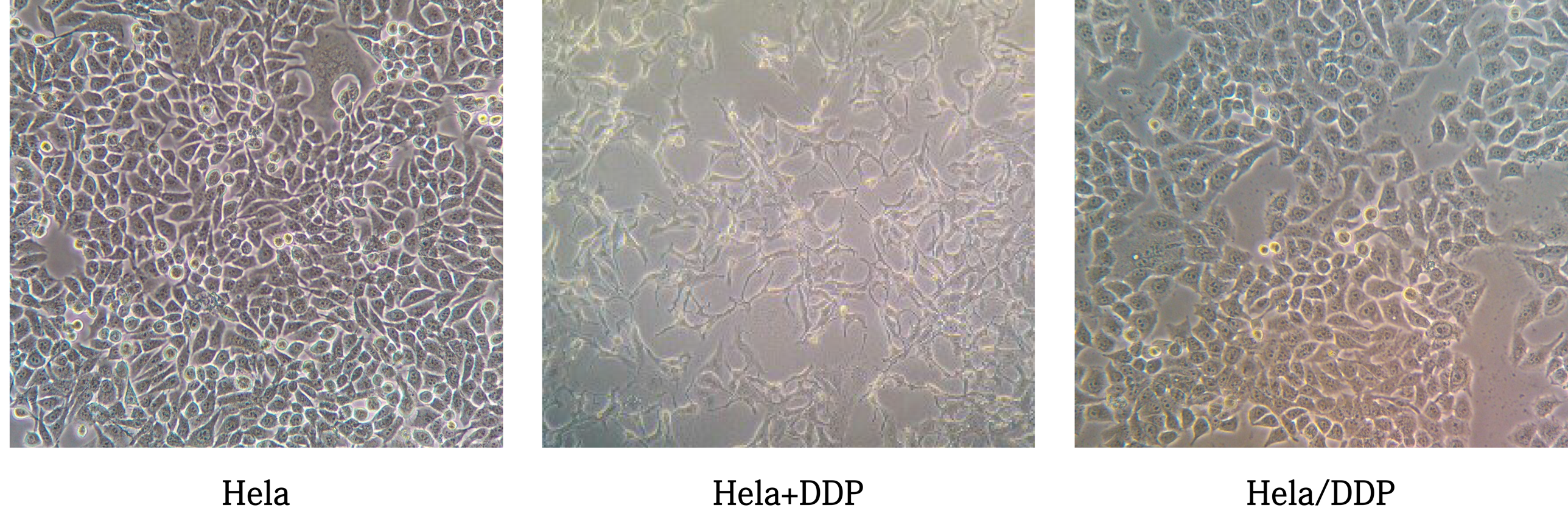

Supplement: Supplementary file 1 — Additional file 1: Figure S1. Morphological observation of HeLa and HeLa/DDP. Magnification, ×100. [file 13008_2019_48_MOESM1_ESM.tif]

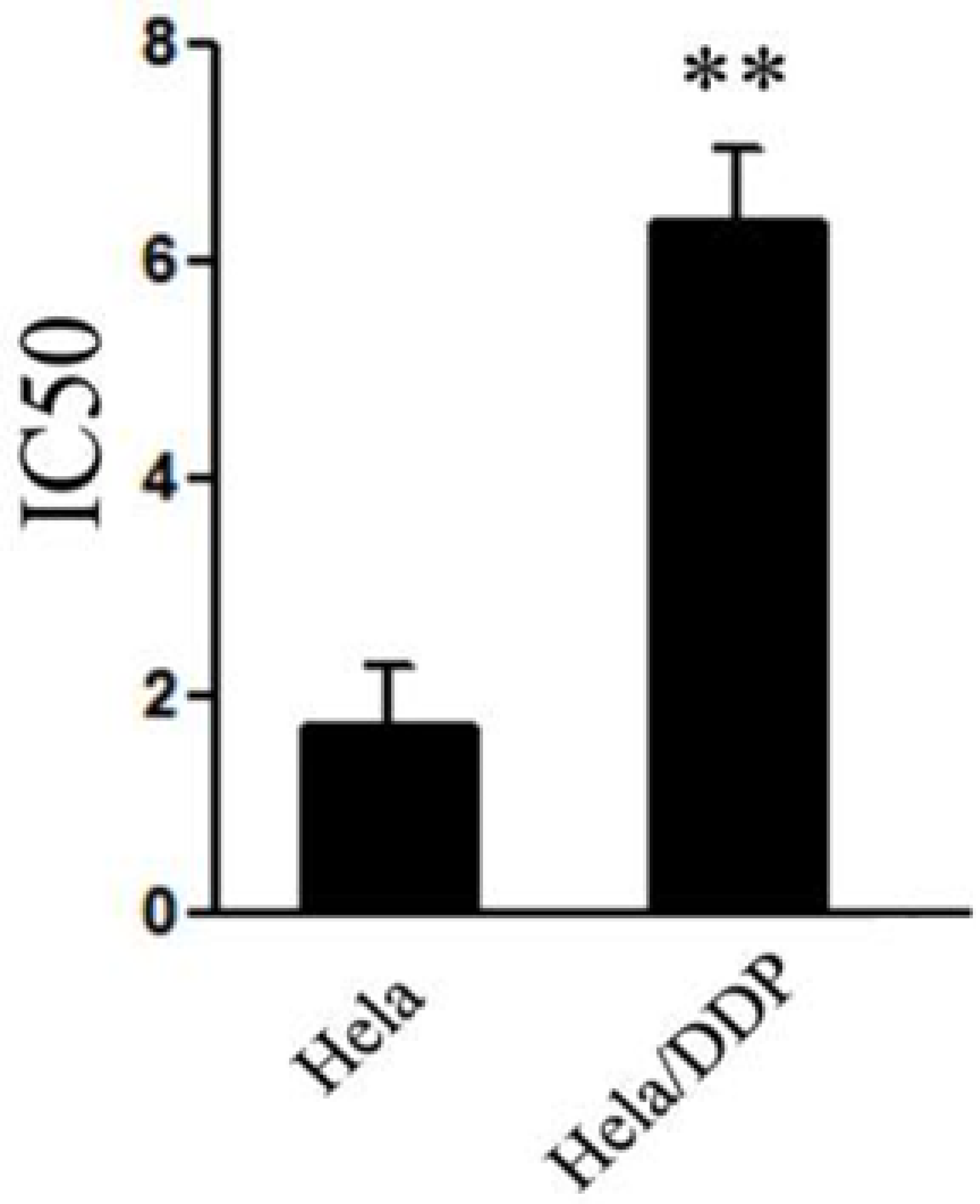

Supplement: Supplementary file 2 — Additional file 2: Figure S2. IC50 of HeLa and HeLa/DDP in the presence of DDP. Values represent mean ± S.E.M. **P < 0.01 versus HeLa group (Student’s t-test). [file 13008_2019_48_MOESM2_ESM.tif]

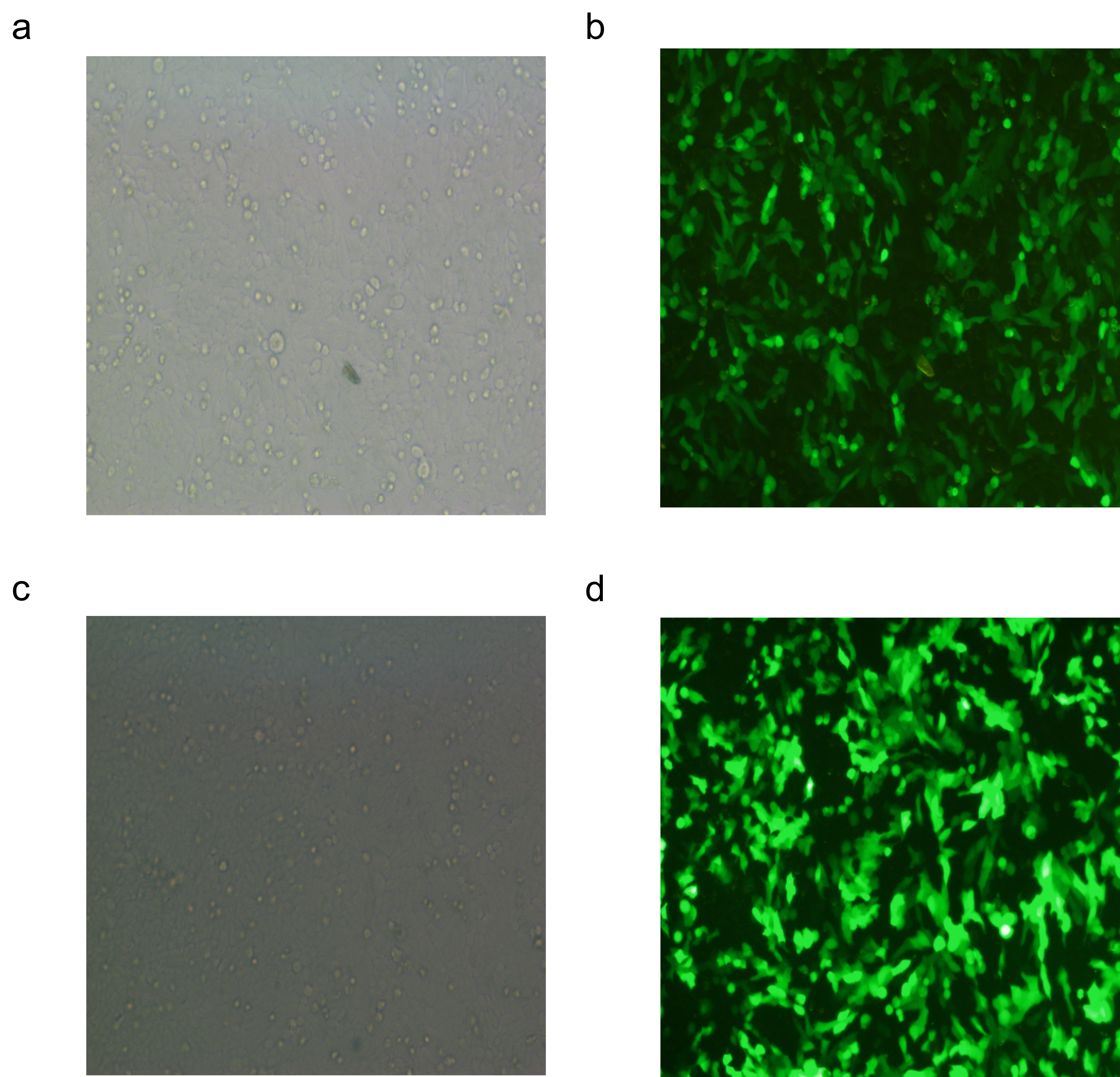

Supplement: Supplementary file 4 — Additional file 4: Figure S4. The expression of GFP under a fluorescence microscope in HeLa/DDP cells after transfection with pEX-2 for 24 h. A. HeLa/DDP cells transfected with pEX-2 P16 (INK4a) under an inverted microscope. B. HeLa/DDP cells transfected with pEX-2 P16 (INK4a) under a fluorescence microscopy. C. HeLa/DDP cells transfected with pEX-2 empty vector under an inverted microscope. D. HeLa/DDP cells transfected with pEX-2 empty vector under a fluorescence microscopy. Magnification, ×100. [file 13008_2019_48_MOESM4_ESM.tif]
